# Muscle Gene Sets: a versatile methodological aid to functional genomics in the neuromuscular field

**DOI:** 10.1186/s13395-019-0196-z

**Published:** 2019-05-03

**Authors:** Apostolos Malatras, Stephanie Duguez, William Duddy

**Affiliations:** 1 Myologie Centre de Recherche, Université Sorbonne, UMRS 974 UPMC, INSERM, FRE 3617 CNRS, AIM, Paris, France; 2 Northern Ireland Centre for Stratified Medicine, Biomedical Sciences Research Institute, C-TRIC, Ulster University, Altnagelvin Hospital Campus, Glenshane Road, Derry/Londonderry, BT47 6SB UK; 3 Department of Biological Sciences, Molecular Medicine Research Center, University of Cyprus, 1 University Avenue, 2109 Nicosia, Cyprus

**Keywords:** Gene sets, Skeletal muscle, Neuromuscular, Functional genomics, Pathway analysis, Functional enrichment, GWAS, Gene expression, Transcriptomics

## Abstract

**Background:**

The approach of building large collections of gene sets and then systematically testing hypotheses across these collections is a powerful tool in functional genomics, both in the pathway analysis of omics data and to uncover the polygenic effects associated with complex diseases in genome-wide association study. The Molecular Signatures Database includes collections of oncogenic and immunologic signatures enabling researchers to compare transcriptional datasets across hundreds of previous studies and leading to important insights in these fields, but such a resource does not currently exist for neuromuscular research. In previous work, we have shown the utility of gene set approaches to understand muscle cell physiology and pathology.

**Methods:**

Following a systematic survey of public muscle data, we passed gene expression profiles from 4305 samples through a robust pre-processing and standardized data analysis pipeline. Two hundred eighty-two samples were discarded based on a battery of rigorous global quality controls. From among the remaining studies, 578 comparisons of interest were identified by a combination of text mining and manual curation of the study meta-data. For each comparison, significantly dysregulated genes (FDR adjusted *p* < 0.05) were identified.

**Results:**

Lists of dysregulated genes were divided between upregulated and downregulated to give 1156 Muscle Gene Sets (MGS). This resource is available for download (www.sys-myo.com/muscle_gene_sets) and is accessible through three commonly used functional genomics platforms (GSEA, EnrichR, and WebGestalt). Basic guidance and recommendations are provided for the use of MGS through these platforms. In addition, consensus muscle gene sets were created to capture the overlap between the results of similar studies, and analysis of these highlighted the potential for novel disease-relevant findings.

**Conclusions:**

The MGS resource can be used to investigate the behaviour of any list of genes across previous comparisons of muscle conditions, to compare previous studies to one another, and to explore the functional relationship of muscle dysregulation to the Gene Ontology. Its major intended use is in enrichment testing for functional genomics analysis.

**Electronic supplementary material:**

The online version of this article (10.1186/s13395-019-0196-z) contains supplementary material, which is available to authorized users.

## Background

### Gene sets in functional genomics

A gene set is a list of genes that share a common feature. Examples include common pathway membership, shared dysregulation in a gene expression study, participation to the same protein complex, or sequence homology (reviewed [1]). Usage of the term overlaps with the concept of a molecular signature, which can take the form of a simple gene list or a more complex data structure, for example, including weightings for each gene. Gene sets, or molecular signatures, have been a key feature of major studies [2–5]. The approach of building large collections of gene sets and then systematically testing hypotheses across these collections is a powerful tool in functional genomics, both in the pathway analysis of omics data (reviewed [6, 7]), and to uncover the polygenic effects associated with complex diseases in genome-wide association study (GWAS) analyses (reviewed [8, 9]).

### Gene set analysis tools and gene set collections

A commonly used approach to pathway analysis is functional enrichment testing. In this approach, the gene set is considered to represent a biological function, and statistical tools are applied to test how this function behaves within the omics dataset under study. Functional enrichment tools often use a standard statistical method called Fisher’s exact test or the hypergeometric test, which evaluates whether the proportion of genes in a results list that also belongs to a given gene set is greater than expected by chance. An example of such a tool is EnrichR, which applies hypergeometric testing across a large variety of gene set collections relating to pathways, ontologies, transcriptional regulation, disease, and other biological themes [10]. The well-known DAVID tool takes a similar approach but also identifies clusters of enriched gene sets based on the proportion of genes that they share with one another [11]. More conceptually subtle approaches make use of the structure of omics data—for example, Gene Set Enrichment Analysis (GSEA) tests the distribution of each gene set within a list of genes that have been ranked based on their degree of differential expression between two experimental groups (from most strongly upregulated to most strongly downregulated) [12]. In GSEA, gene sets having their members clustered disproportionately (as determined by permutation-based statistics) within one region of the list are considered to be enriched, and a weighting is used to score more highly those regions representing strong upregulation or downregulation (i.e. the two ends of the distribution). GSEA and similar tools have recently been systematically compared [13]. Another tool, WebGestalt, provides multiple analytical approaches within a single framework [14].

Gene set analysis of GWAS data is a maturing field [8], with tools such as MAGMA [15], MAGENTA [16], and others enabling the discovery of genome-wide pathway associations in a number of diseases [16–18]. Whereas classic GWAS seeks to identify associations to one or more single nucleotide polymorphisms (SNPs), gene set approaches summarize SNPs by gene and then by gene set. This improves the power to detect statistically significant associations both because collapsing individual SNPs into gene sets results in fewer statistical tests performed and because individual weak effects can be combined to produce a strong association signal [9].

A widely used collection of gene sets is the Molecular Signatures Database, MSigDB [19, 20], which is divided into eight major collections. These include curated gene sets from pathway databases such as KEGG [21], the Gene Ontology (GO) [22], and genes with shared regulatory motifs or chromosomal positions. Importantly, in the context of the present work, MSigDB also includes collections of genes having shared dysregulation in cancer or immunologic gene expression studies. These last two collections, ‘oncogenic signatures’ and ‘immunologic signatures’ have been created by systematic analyses of relevant datasets from the Gene Expression Omnibus (GEO) [23], enabling researchers to compare later transcriptional datasets across hundreds of previous studies [24], and thereby contributing to leading publications in their field [25, 26].

### Gene sets for functional genomics in the study of skeletal muscle tissue and neuromuscular pathology

Functional genomics is integral to the current study of skeletal muscle tissue and neuromuscular pathology, as evidenced by the vast quantities of omics data now generated by researchers in this field. Considering RNA expression alone, a simple search of ‘skeletal AND muscle’ in the ArrayExpress database returns more than 1000 separate experimental studies of gene/transcript and microRNA expression [27]. These studies frequently make use of pathway analysis approaches, but this is done without the aid of gene set collections of the subject-specific type that have been beneficial to researchers in the fields of oncology and immunology.

In prior work, we have extracted gene sets from published muscle gene expression data and applied these for analytical purposes in three previous studies: (1) In a study of human myotubes from old compared to young subjects, we found a similar profile of dysregulation to that observed in previous gene expression studies of myoblast differentiation, despite that the fusion index of old myotubes was unaffected—this led to the discovery of a failure of re-quiescence in elderly myoblast cultures, and the identification of SPRY1 methylation as an underlying mechanism, with resultant loss of the stem cell pool having a potential role in sarcopenia [28]. (2) In an analysis showing that the procedure of hTERT/cdk4 immortalization did not impact on the skeletal muscle characteristics of human myoblasts, we studied the expression levels of consensus sets of genes that were up- or downregulated consistently across multiple studies of muscle differentiation [29]. (3) A collection of muscle gene sets was also used to aid in the characterization of a murine model of Annexin A2 knockout, to better understand the role of this protein in sarcolemmal repair and dysferlinopathy [30]. These studies demonstrated the utility of muscle-specific gene sets for functional genomics analyses.

### Muscle Gene Sets

Here, we report the creation of the Muscle Gene Sets (MGS) resource (sys-myo.com/muscle_gene_sets), a collection of gene sets extracted from expression studies of skeletal muscle cells and tissues, and a smaller number of cardiac studies. These relate to various aspects of muscle molecular physiology and pathology, including myopathies, cardiomyopathies, metabolism, exercise, ageing, development, regeneration, and others. The MGS can be accessed through the site itself and also through three analytical tools—Enrichr, MSigDB/GSEA, and WebGestalt. We also generated consensus gene sets, identifying genes that are commonly dysregulated in the same experimental comparison across multiple different studies.

## Methods/implementation

### Microarray data collection

Data were downloaded from public gene expression resources, ArrayExpress and Gene Expression Omnibus (GEO). Although ArrayExpress mirrors GEO, the mirroring is not perfect, so we searched both repositories for striated muscle (skeletal and cardiac), cells, and cell line experiments. In this initial screening, we found that the most abundant microarray chips used for muscle-related experiments were Affymetrix Human Genome U133 Plus 2.0 GeneChip (GPL570 GEO platform or A-AFFY-44 ArrayExpress ID) for human and Affymetrix Mouse Genome 430 2.0 GeneChip (GPL1261 GEO platform or A-AFFY-45 ArrayExpress ID) for murine samples. In order to maintain a homogenous analytical approach, we narrowed down our next search to these two platforms, which represent about 50% of all muscle arrays on both repositories.

We searched ArrayExpress and GEO using the following string: *(muscle(s) OR myoblast(s) OR myotube(s) OR myofiber(s) OR cardiomyocyte(s) OR myocyte(s) OR heart(s) OR C2C12 OR HSMM OR HL1 OR G8 OR SOL8) AND A-AFFY-44* for human and *(muscle(s) OR myoblast(s) OR myotube(s) OR myofiber(s) OR cardiomyocyte(s) OR myocyte(s) OR heart(s) OR C2C12 OR HSMM OR HL1 OR G8 OR SOL8) AND A-AFFY-45* for mouse organisms. However, it is getting more and more usual for researchers to use an alternative probe to gene mapping file, called Chip Description File (CDF), than the original from Affymetrix, for better probe to probeset and probeset to gene targeting accuracy. GEO and ArrayExpress assign a unique GPL or ID key respectively for each of the alternative GEO platforms or ArrayExpress IDs while microarray chips remain the same. In order to find the alternative platforms, GEO provides a list of them on the original platform GPL, but this is not well maintained and many are missing. A more certain way to identify them is to search on ArrayExpress (which is manually curated) for alternative IDs. On ArrayExpress’s browse page (https://www.ebi.ac.uk/arrayexpress/arrays/browse.html), we searched for *U133 Plus 2.0*, MG 430 2.0 and retrieved all the alternative GEO platforms and IDs to A-AFFY-44 (GPL570) for human and to A-AFFY-45 (GPL1261) for mouse.

Next, we parsed their MIAME [31] conformed metadata by text mining and confirmed them manually, selecting only those pertinent to muscle research. We excluded all series that did not include the raw CEL files (Affymetrix fluorescence light intensity files) in order to homogenize the data even further by preprocessing all raw files with a robust pre-processing and data analysis pipeline [32].

### Affymetrix microarray quality assessment

Despite that the arrays are published and have already passed quality controls (QCs), these QC steps have been applied differently by different authors. For this reason, we performed a global quality control using a battery of Bioconductor [33, 34] packages: ‘simpleaffy’ [35], ‘affyQCReport’, and ‘affyPLM’ [36], using the MAS 5.0 algorithm [37] and the Affymetrix default Chip Description File (CDF). We used the Affymetrix chip embedded single array quality metrics for each sample, such as average background, scale factor, the percentage of genes called present, and 3′ to 5′ RNA hybridization ratios for β-actin and GAPDH. We also used two multi-array quality metrics for each series, Normalized Unscaled Standard Error (NUSE) and Relative Log Expression (RLE). As a general guideline, we followed Affymetrix recommended thresholds: differences in average background per sample not higher than 20, scale factor within a threefold change of one sample to another, no higher than 10% difference of percent present genes and 3′ to 5′ ratio threshold of GAPDH to 1.25 and β-actin to 3. Also, the NUSE boxplots should be centered on 1 with bad-quality samples ranging above 1.1. Samples were also deemed as low quality if they had globally higher spread of NUSE distribution than others. Because it is assumed that most probes are not changed across the arrays, the ratio of probeset expression and the median probeset expression across all samples of a series are expected to be around 0 on a log scale. The RLE boxplots presenting the distribution of these log ratios should be centered near 0 and have similar spread with low-quality samples having a spread higher than 0.2. Arrays that had extreme values or were above our set thresholds on the combined QCs were not used for any further analysis. In total, we removed 160 human and 122 mouse samples. In our case, percent present and RLE performed better than the other metrics, as also reported by McCall et al [38].

### Data normalization

Pre-processing algorithms, usually termed normalization algorithms, are three-step processes: background correction, normalization, and probe summarization. The arrays that passed quality controls were pre-processed with the Robust Multi-array Average algorithm [39], with default parameters except for the CDFs that were downloaded from BrainArray ENSG version 20.0.0 [40].

### Probes to gene mapping

The microarray Affymetrix GeneChips we collected to create the MGS are the most abundantly used chips for human and mouse microarray experiments. However, their selection of probes relied on early genome and transcriptome annotation (2003–2004) which is significantly different from our current knowledge. Most of the genes on the microarray chips are usually represented by a few probesets, and in many cases, a probeset could target multiple genes. Probesets that target a gene could exhibit wildly different expression levels making downstream analysis challenging. Dai et al. had foreseen these limitations and created the BrainArray portal [40] where they reorganize probes with up-to-date genome, cDNA, and single nucleotide polymorphisms (SNPs) information in order to create a more accurate and precise CDF, which is widely used in gene mapping [41]. BrainArray’s CDF is updated annually with most microarray algorithms and tools supporting its CDF by default.

### Allocation of samples to comparisons of interest

Sample meta-data were mined from GEO or ArrayExpress and manually inspected to allocate samples to comparisons of interest, as well as to name each comparison. During this manual process, reference was frequently made to the original GEO pages for individual samples, and often back to the publication associated to the data, in order to confirm sample designations and points of methodology. In cases where the relevant characteristic of samples could not be clearly established, the comparison was not used. Selected comparisons were taken forward to differential expression analysis.

### Differential expression analysis and gene mapping

We used the ‘limma’ package [42, 43] for differential expression analysis. We included into gene sets all genes with Benjamini-Hochberg FDR adjusted *p* value < 0.05 for each experimental comparison, up to a maximum of 300 genes, taking the top 300 after ranking by the significance of differential expression (Limma’s B statistic). Before the eBayes step, we also removed 25% of the genes that had the lowest average expression values. To map Ensembl gene IDs to gene symbols, we used Ensembl BioMart [44]. We extracted the required information from GRCh38.p5 assembly for human and GRCm38.p4 assembly for mouse. Following the standard of MSigDB, standard gene names were used that are approved by the Human Gene Nomenclature Committee (HGNC) and Mouse Genome Informatics (MGI) groups.

### Batch effect correction

For batch effect identification and correction, we used the surrogate variable analysis (sva) algorithm [45] from the ‘SVA’ Bioconductor package [46]. We used the ‘leek’ method to detect the number of surrogate variables, if present, but we also set a limit of up to two surrogate variables to avoid overcorrecting the data. The sva algorithm found and corrected technical variation in 256 out of a total of 578 experiments.

### Consensus set enrichment analysis

Genes of selected consensus sets were tested for enrichment against the Gene Ontology sections, biological processes, and cell compartments, using the EnrichR tool.

### Contents of the MGS website

The full downloadable MGS collection includes 1517 gene sets: 1156 gene sets from the current work, which for completeness include 245 empty gene sets for which no significantly differentially expressed genes were identified; 122 gene sets derived from post-2005 studies of myoblasts and myotubes, referenced in our previous work [29]; 185 gene sets extracted from a previous meta-analysis of early muscle microarray data (pre-2005; [47]); and 54 gene sets identified by searching for muscle-relevant terms within the MSigDB collections (mostly comprising muscle-related pathways from Reactome or Biocarta databases and omitting any gene sets that could duplicate those created in the present analysis).

## Results

### Creation of gene sets

**Fig. 1:**
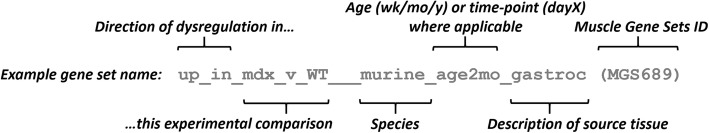
Naming convention for Muscle Gene Sets. Each name was chosen to be both succinct and readily understandable. This was not an automated process—consideration was given to the name of each gene set. The first segment, before the triple underscore, has the generic form ‘up_in_Group1_v_Group2’ or ‘down_in_Group1_v_Group2’, referring to genes that were up- or downregulated in the comparison of group 1 (e.g. mdx) to group 2 (e.g. WT), for which ‘up’ indicates greater expression in group1 compared to group2, and ‘down’ means lesser expression in group1. Following the triple underscore, species name is then given, then age/timepoint and/or tissue description and/or gender (in any order). Finally, each gene set is given a MGS ID number. List of time abbreviations used: h = hour(s); d = day(s); wk. = week(s); mo = month(s); y = year(s). List of other abbreviation conventions used (ordered by appearance in the complete MGS gmt file): ctl = control; WT = wild-type; gastroc/gastr = gastrocnemius muscle; DMD = Duchenne muscular dystrophy; quad = quadriceps muscle; skel = skeletal; dysf = dysferlinopathy; EDMD = Emery-Dreifuss muscular dystrophy; EDL = extensor digitorum longus muscle; TA/tib_anterior = tibialis anterior muscle; diff = differentiation/differentiated (of myotubes); prim = primary cells; vast_lat/vastus_lat = vastus lateralis; KO = knock-out; mir = microRNA. Some study-specific abbreviations are used, which are assumed to be understandable from context or occasionally requiring reference to the source GEO entry indicated in the information column of the gmt file

Following a systematic survey of public gene expression data repositories, we downloaded raw expression data from 302 studies of muscle gene expression, including 4305 separate samples. After robust pre-processing through a standardized data analysis pipeline, 282 samples were discarded based on a battery of rigorous global quality controls. From among the remaining studies, 578 comparisons of interest were identified by a combination of text mining and manual curation of the study meta-data. For each comparison, significantly dysregulated genes (FDR *p* < 0.05) were identified and divided into two lists of up to 300 each: those that were most significantly upregulated and those that were most significantly downregulated. Each of these 1156 lists was considered a Muscle Gene Set and was given a name tag intended to be both succinct and readily understandable (Fig. 1).

The primary format in which the MGS collection is stored is as a gmt file of the type used by MSigDB [19] and GSEA [12]. This is a tab-delimited plain text format in which each gene set is represented by a new line. The name of the gene set is given first, followed by an information field that includes an identifier (usually a GEO or ArrayExpress series number) linking back to the original data source. Member genes of the gene set are then listed.

### Content of the MGS collection

**Fig. 2:**
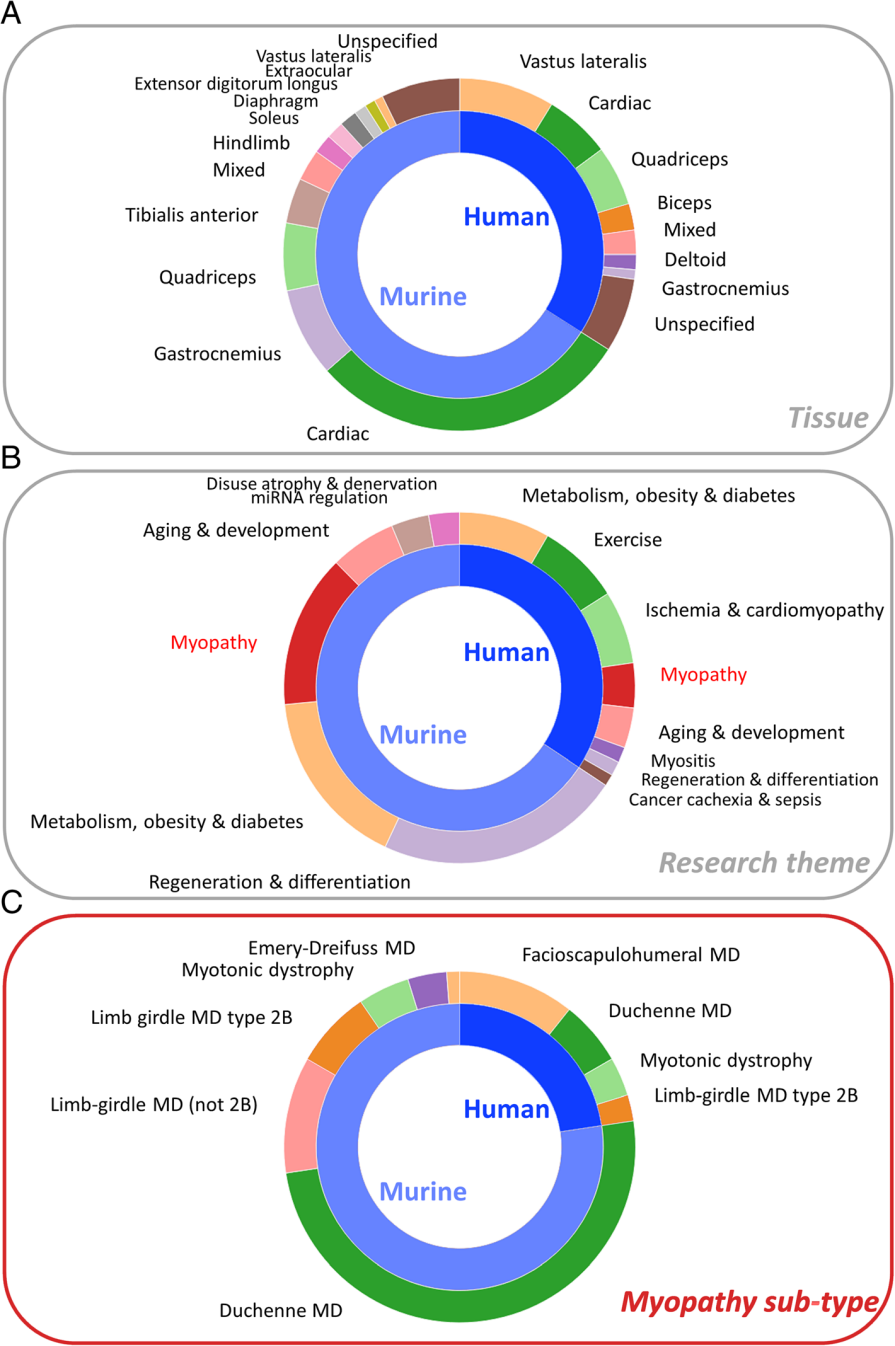
Proportional composition of the MGS collection broken down by tissue type, research theme, and myopathy sub-type, for human and murine species. **a** Tissue types. Shown are all tissue categories containing 10 or more gene sets. ‘Mixed’ indicates that the comparison is between different tissue types (e.g. gastrocnemius vs vastus lateralis). The ‘unspecified’ category indicates gene sets from studies in which the specific muscle tissue was not given in the published work. **b** Research themes. Shown are all themes containing 10 or more gene sets. **c** Myopathy sub-types. These are sub-categories of the myopathy set in **b**. All are shown

Each muscle gene set represents either the up- or downregulated genes from a single comparison within a gene expression study. The studies included human or murine muscle tissues and cells, and the comparisons are made between different muscle tissues, ages and developmental stages, pathologies, experimental treatments, and genetic interventions. A breakdown of the composition of the MGS by tissue, research theme, and type of myopathy is shown in Fig. 2.

Murine studies accounted for 65.9% of muscle gene sets, being about twice as common as human studies (34%), and cardiac-related gene sets accounted for a large proportion (44.7%) of these (Fig. 2a). Of skeletal muscle gene sets, murine samples were frequently (22.4%) derived from the gastrocnemius, which was relatively rarely studied (3.2%) in humans. Conversely, the vastus lateralis was the most common origin of human muscle gene sets, while this muscle is not much studied in mice. We also tagged gene sets according to specific research themes (Fig. 2b). Many murine gene sets were related to regeneration and/or differentiation, largely due to studies of C2C12 myoblasts. A substantial proportion of both human (23.9%) and murine (24.7%) gene sets relates to studies of metabolism, obesity, and/or diabetes, while gene sets relating to exercise are almost all derived from human studies. Myopathies accounted for 11.8% and 21.1% of human and murine gene sets, with other common research themes including ageing and development, miRNA regulation, disuse atrophy, cancer cachexia, and sepsis.

Among myopathy-related gene sets (Fig. 2c), precisely 50% are derived from a large number (~ 40) of studies of the murine model (mdx) of Duchenne muscular dystrophy (DMD). DMD also accounted for 26% of human myopathy gene sets, although in humans facioscapulohumeral muscular dystrophy (FSHD) had the most commonly studied gene expression, accounting for 47% of human myopathy gene sets. Limb-girdle muscular dystrophy type 2B (LGMD2B) and the myotonic dystrophies (DM1 and DM2) account for around 10% each of human and murine myopathy gene sets, while a small number of gene sets were derived from murine studies of other limb-girdle muscular dystrophies and of Emery-Dreifuss muscular dystrophy (EDMD).

### Consensus Muscle Gene Sets

Since comparisons of similar conditions were sometimes carried out in multiple different published studies, we created high-confidence consensus sets containing only those genes that were consistently dysregulated in the same direction for the same comparison across those studies. For this purpose, we manually identified 250 gene sets belonging to 14 different comparisons (Table 1), each comparison having a minimum of 8 gene sets (4 upregulated and 4 downregulated). For each comparison, we identified genes that were consistently upregulated, downregulated, or dysregulated (and in the same direction). For each of these 3 cases (up, down, and same), we created consensus sets for genes shared by at least 30%, 50%, or 70% of the gene sets, giving a total of 9 consensus sets for each comparison.

**Table 1:** Consensus muscle gene sets

Category	Consensus set name (indicating tissue type and comparison)	# Muscle Gene Sets
Aging and development	HumanSkelMusc_Aging_v_Young	16 (8 up, 8 down)
	MurineSkelMusc_12orMoreMonths_v_1to5Months	8 (4 up, 4 down)
	MurineSkelMusc6WkOrOlder_Mdx_v_Healthy	36 (18 up, 18 down)
Differentiation	MurineMyotube_12to24hDiff_v_Undiff	14 (7 up, 7 down)
	MurineMyotube_2orMoreDaysDiff_v_Undiff	38 (19 up, 19 down)
	MurineMyotube_9hOrLessDiff_v_Undiff	18 (9 up, 9 down)
Disuse	AnySpeciesSkelMusc_AtrophyDisuseOrInactivated_v_Control	28 (14 up, 14 down)
Exercise	HumanSkelMusc_1DayOrLessAfterExercise_v_Before	18 (9 up, 9 down)
	HumanSkelMusc_8wkOrMoreAfterResistanceTraining_v_Before	12 (6 up, 6 down)
Metabolism	HumanSkelMusc_Type2_Diabetes_v_Healthy	10 (5 up, 5 down)
	MurineSkelMusc_HighFatDiet_v_Control	18 (9 up, 9 down)
Myopathy	HumanOrMurineSkelMusc_Dysferlinopathy_v_Control	12 (6 up, 6 down)
	HumanSkelMusc_DMD_v_Healthy	8 (4 up, 4 down)
	MurineSkelMusc_Calpainopathy_v_Healthy	14 (7 up, 7 down)

In general, consensus sets consisted of genes that would be expected according to previous literature. For example, functional analysis of 39 consensus genes that were dysregulated by a high-fat diet in murine skeletal muscle (Additional file 1: Figure S1A), drawn from 18 gene sets from 7 different published studies, showed upregulation of fatty acid oxidation and the mitochondria, these two processes being driven by upregulated mitochondrial genes ACADVL (very long-chain specific acyl-CoA dehydrogenase), ACAA2 (3-ketoacyl-CoA thiolase), ECI1 (Enoyl-CoA delta isomerase 1), ACADM (medium-chain specific acyl-CoA dehydrogenase), HADH (hydroxyacyl-coenzyme A dehydrogenase), DECR1 (2,4-dienoyl-CoA reductase), and ACAT1 (Acetyl-CoA acetyltransferase). Change to expression levels of fatty acid oxidation genes was also the major process enriched 1 day following exercise in human skeletal muscles (Additional file 1: Figure S1B).

Expectedly, myotube differentiation up to 9 h consistently involved changes in the expression patterns of genes involved in chromatin remodelling (Additional file 1: Figure S1C), which was succeeded at 12–24 h (Additional file 1: Figure S1D) by changes in cell cycle gene regulation, with significant but less pronounced enrichment of sarcoplasmic reticulum (SR) and calcium channel genes. After 2 or more days of maturation, the dominant dysregulated process (compared to day 0) was muscle contraction (Additional file 1: Figure S1E).

More notably, in Duchenne muscular dystrophy compared to healthy controls, alongside expected observations such as downregulation of the dystrophin-associated protein complex and upregulation of extracellular matrix components expected as a result of fibrosis, what may be surprising is the extent to which lysosomal genes were consistently found to be upregulated (Additional file 1: Figure S1F). Of 84 consensus genes upregulated in 50% or more of DMD vs healthy comparisons in human skeletal muscle, 11 lysosomal genes were present (enrichment FDR *p* value 0.00002). Four of these genes overlapped with the upregulated extracellular matrix genes, but enrichment of the lysosome was still significant (FDR < 0.0001) when these 4 genes were omitted from the analysis. Very similar enrichment results emerged from consensus genes in the mdx murine model of DMD, with strong enrichment of the lysosomal/ER lumen (Additional file 1: Figure S1H). This was driven by a different set of genes in mouse than in humans; the only lysosomal gene shared between human and murine consensus sets was LGMN, which encodes Legumain, a protein which hydrolyzes asparaginyl bonds in lysosomal protein degradation.

Other comparisons for which consensus sets were identified included 6 studies of human and murine dysferlinopathy versus healthy, for which neutrophil-mediated immunity and vacuolar/lysosomal lumen were the most strongly dysregulated process and component, respectively, each being upregulated (Additional file 1: Figure S1I). Ageing compared to young human skeletal muscle across 8 studies resulted in changes to mitochondrial ATP synthesis coupled proton transport and regulation of RNA transcription and, less expectedly, changes to regulation of neuron apoptotic process, although this latter enrichment was due to only 2 genes (PRNP and FOXO3) (Additional file 1: Figure S1J). The most frequently recurring impact of resistance training in 6 human studies was changes to expression of collagens, which drove enrichment of both extracellular matrix reorganization and of endoplasmic reticulum lumen (Additional file 1: Figure S1K). Changes to expression of collagens were also the main consistent feature of murine skeletal muscle ageing in 4 studies comparing 12 or more months against 1 to 5 months old (Additional file 1: Figure S1L).

Several attempts to generate consensus sets failed due to low overlap between the results of different studies. No genes were dysregulated in the same direction in 2 or more out of 5 studies of human type 2 diabetes vs healthy (Additional file 1: Figure S1M). Across 7 studies of murine calpainopathy, only 12 genes were consistently dysregulated in 30% or more of the studies, and these 12 genes were not significantly enriched for any biological process or cell component (Additional file 1: Figure S1N). Only 4 genes were consistently dysregulated in 30% or more of 14 studies of muscle atrophy, disuse, or inactivation, although here we did not attempt to generate consensus lists for human and murine separately (Additional file 1: Figure S1O).

### Accessibility and implementation of the Muscle Gene Sets in functional genomics analysis

The MGS repository, including the complete collection of MGS as well as, separately, the consensus sets, is available for download in .gmt format from our Sys Myo site (at https://www.sys-myo.com/muscle_gene_sets/). We have also worked with other developers to include the MGS in two online functional genomics analysis tools, EnrichR [10] and WebGestalt [14].

The MGS is currently the last listed collection in EnrichR’s ‘Crowd’ category. Using EnrichR, it is very straightforward to submit a query list of gene names of interest and carry out functional enrichment testing to determine whether the query list is enriched for any of the muscle gene sets. This facilitates rapid screening of any list of genes against the genes that were dysregulated in each of the previous muscle studies of gene expression.

Analysis using the MGS in WebGestalt is achieved by selecting either hsapiens or mmusculus as the organism of interest, the Overrepresentation Enrichment Analysis (ORA) or Gene Set Enrichment Analysis (GSEA) as the method of interest, and selecting community-contributed functional database. The MGS collection is then selectable, and a list of genes can be uploaded for enrichment testing against the MGS.

The .gmt format in which we provide the MGS gene set collection was originally developed for the GSEA tool [12] (http://software.broadinstitute.org/gsea/), and a large variety of gene set collections are available in this format for download from the Molecular Signatures Database (MSigDB [19]). MSigDB are now listing gene sets from community contributors, of which MGS is the first listed (http://software.broadinstitute.org/gsea/msigdb/contributed_genesets.jsp). Enrichment testing can be carried out on the MGS collection using the GSEA software by uploading the MGS .gmt file as a local gene matrix from within the GSEA software’s gene set database selection dialogue. We note that it is also possible to concatenate .gmt files using a text editor or scripting language, and it can be of interest to compare, for instance, enrichment results of Gene Ontology terms against those of the MGS. To facilitate deeper functional interpretation of the results, the output from GSEA can be visually displayed using the Enrichment Map plugin [48] for Cytoscape, for example, to examine the overlap of enriched MGS with enriched GO terms, as we have done previously [28, 30].

## Discussion

According to developed principles for the organization of gene sets [1], MGS are phenotypic-level gene sets in which genes share actual connections in the form of differential expression in the same transcriptomic comparison. The MGS resource can be used to investigate the behaviour of any list of genes across > 1100 previous comparisons of muscle conditions, to compare previous studies to one another, and to explore the functional relationship of muscle dysregulation to the gene ontology. Its major intended use is in enrichment testing for functional genomics analysis, for which purpose it has been made accessible through three commonly used analytical tools (GSEA, EnrichR, and WebGestalt).

### Optimal usage

Various statistical approaches are used for gene set enrichment analysis, and their performances have been evaluated and usage guidelines established [49]. GSEA is an active field in which new methods are being developed, such as to identify gene sets that are specifically enriched in one experiment among a large set of experiments [50]. An important advantage of gene sets in expression pattern analysis is that they enable the identification of gene groups whose constituents show subtle but coordinated expression changes, which might not be detected by the usual individual gene analysis [51]. We consider that the GSEA algorithm is particularly suited to this as it can detect subtle shifting of a gene set within a differential expression profile, and because it profiles across the entire expression matrix, it is not dependent on an arbitrary statistical cut-off to distinguish dysregulated from unaffected genes. Of the different methods to use the MGS, we consider that a very powerful approach is to concatenate the MGS with the Gene Ontology (or another functional annotation such as Reactome or KEGG pathways), then test a ranked query gene list against this concatenated gene set collection using the GSEA tool, and visualize the results using the enrichment mapping plugin for Cytoscape. This approach, which we have adopted in previous work [28, 30], makes it possible not only to identify the overlap of query genes with previous muscle studies, but also to understand the overlap of previous muscle studies with elements of the Gene Ontology, including biological processes and cell compartments, which greatly aids in the understanding of what the enrichment results mean, helping to interpret them in their biological context.

However, for rapid enrichment testing of a gene list against the MGS collection, without deeper analysis of overlap between gene sets, both EnrichR and WebGestalt are very convenient and robust resources and are preferable in many instances due to their speed and relative ease of use. It should also be noted that EnrichR’s *z*-score approach is an improvement over the standard Fisher’s/hypergeometric enrichment test and that the WebGestalt pipeline offers multiple implementations of enrichment testing each with specific advantages.

### Consensus muscle gene sets

In creating the MGS resource, we have observed that many of the hundreds of published skeletal muscle gene expression studies have made similar experimental or pathology-relevant gene expression comparisons or identified overlapping sets of dysregulated genes. The MGS provides an opportunity for in-depth study of this overlap, which may reveal new insights into skeletal muscle pathology. We have previously used a consensus MGS to study differentiation in immortalized myoblasts [29].

For instance, an intriguing observation was that lysosomal genes were the most consistently upregulated cell compartment across studies of DMD compared to healthy skeletal muscle. This was true for consensus sets of both human DMD and the murine Mdx model of DMD. Among the publications associated to these data, the lysosome was discussed only rarely [52], with attention usually focused on more general inflammatory/immune response or on other pathways such as calcium homeostasis and fibrosis (extracellular matrix), although upregulation of specific lysosomal genes was reported in 2 human studies (lysosomal acid lipase, cholesteryl ester hydrolase (LIPA) and lysozyme [53]; lysosomal-associated transmembrane protein 5 (LAPM5) [54]) and in 2 murine studies (Lysosome M [55]; Lysozyme [56]). Since the DMD and Mdx consensus sets are drawn from studies of whole muscle tissue, a trivial explanation could be the infiltration of immune cells that is associated with muscle wasting in the disease, and indeed, we observe an enrichment of T cell proliferation and lymphocyte migration among the consensus upregulated genes. However, it has been observed that lysosomal-associated membrane protein, LAMP1, and other vesicular trafficking proteins are oversecreted from DMD myotubes and that disturbance of protein export may make a low-level chronic contribution to DMD pathology [57].

Lysosomal upregulation was also a feature of the dysferlinopathy consensus MGS. Upregulation of the lysosome has not been reported in the literature specifically for dysferlinopathy, although it has been reported as a common feature of muscular dystrophies in a previous gene expression meta-analysis [58], and previous studies of dysferlinopathy have reported dysregulation of specific lumen proteins such as Cathepsin K [59] and Cathepsin S [60]. Similarly to DMD, this could be due to immune cell infiltration or to gene expression changes in the muscle fibres themselves.

### Limitations and perspectives

This analysis captures and summarizes more than 300 studies of muscle gene expression published in the period 2005–2016, and the full MGS collection available for download extends back from 2005 thanks to a previous meta-analysis by Jelier et al. [47]. This does mean that more recent studies are not yet represented. We estimate there could be upwards of 50 new studies since we completed our data curation step. Included among these are a rising number of RNA-Seq-based expression analyses. It will be important to capture these newer studies in future work. The inclusion of RNA-Seq studies will be difficult for the immediate future as they require large computational resources. Given current hardware costs, re-analysis is possible by a small team with limited resources on a per-study basis, but would be challenging to carry out on a systematic multi-study basis, if the analysis goes back to using raw read data. By comparison, it is possible to process very large numbers of microarray samples with only moderate computational demands. We welcome efforts to automate or semi-automate gene set extraction [61–63], which can help in this task, but we note that these require the use of expression matrices that have been supplied to GEO/ArrayExpress by the team making the data submission and therefore depend on the data processing steps carried out by each team, whereas we chose to re-analyse raw probe intensities (.CEL files). We considered this to be of strong importance in order to standardize data processing and quality control. Interesting methods are also being developed for the extraction of gene sets from literature text mining [64].

Gene sets for pre-2005 muscle studies are included in the full downloadable MGS collection. These were obtained from a very thorough previous review and meta-analysis [47] in which gene sets were extracted from publication tables and other sources. The raw data were not re-analysed directly by the authors of the meta-analysis, and a variety of different technological platforms were used by the authors of each study. This is understandable because, especially at that time, microarray platforms were considerably more disparate, so it would have been less useful to focus the meta-analysis on the market-leading platform, as we have been able to do, and normalizing across technological platforms is notoriously difficult [65, 66]. Although we believe there is value in the comparison across these pre-2005 studies collectively (and this is supported by the very interesting meta-analysis that was previously performed on them), due to improvements in microarray technology since 2005, we would urge caution in close interpretation of any given gene from these studies, or any given study treated alone.

We did not extend our analysis to proteomic data—this would be interesting in future work as protein levels more closely reflect the activities of biological pathways. It would also be interesting to systematically cross-relate gene expression to proteomic studies in order to identify which muscle transcripts consistently serve as faithful markers of the proteins that they encode.

Future work could allow selection by keyword, or automated subsetting of the MGS, similarly to that facilitated by the MSigDB resource for its current collections, and perhaps by incorporation of the MGS into the MSigDB, if that becomes an option that MSigDB would provide to community contributors.

## Conclusions

The MGS resource has multiple applications as a research aid in the study of muscle physiology and disease and should be a useful and versatile tool for functional genomics analysis in the neuromuscular field.

## Additional file

Additional file 1: Summary information for consensus muscle gene sets. (PDF 936 kb)

## Abbreviations

CDF: Chip Description File
DM1/DM2: Myotonic dystrophies types 1 and 2
DMD: Duchenne muscular dystrophy
EDL: Extensor digitorum longus muscle
EDMD: Emery-Dreifuss muscular dystrophy
FDR: False discovery rate
FSHD: Facioscapulohumeral muscular dystrophy
GEO: Gene Expression Omnibus
GSEA: Gene Set Enrichment Analysis
GWAS: Genome-wide association study
KO: Knock-out
LGMD2B: Limb-girdle muscular dystrophy type 2B
MGS: Muscle Gene Sets
MIAME: Minimum information about a microarray experiment
MSigDB: Molecular Signatures Database
NUSE: Normalized Unscaled Standard Error
QC: Quality controls
RLE: Relative Log Expression
SNP: Single nucleotide polymorphism
TA: Tibialis anterior muscle
WT: Wild-type
