# Supplementary material for: Muscle Gene Sets: a versatile methodological aid to functional genomics in the neuromuscular field
Source: Skelet Muscle. 2019 May 3;9:10. doi: 10.1186/s13395-019-0196-z (PMC6498474; doi:10.1186/s13395-019-0196-z)
Supplement: Supplementary file 1 — Summary information for consensus muscle gene sets. (PDF 936 kb) [file 13395_2019_196_MOESM1_ESM.pdf]

Consensus gene set name: MurineSkelMusc_HighFatDiet_v_Control
Number of gene sets drawn from: 18 (9 up, 9 down)
EnrichR test carried out on consensus proportion of: 30% Up: 39 genes (4.08%)

Biological processes

Index	Name	P-value	Adjusted p-value	Z-score	Combined score
1	fatty acid oxidation (GO:0019395)	5.823e-14	1.153e-11	-1.82	55.51
2	fatty acid catabolic process (GO:0009062)	5.145e-13	6.791e-11	-1.83	51.70
3	fatty acid beta-oxidation (GO:0006635)	5.823e-14	1.153e-11	-1.53	46.53
4	fatty acid beta-oxidation using acyl-CoA dehydrogenase (GO:0033539)	0.000003772	0.0003734	-2.33	29.13
5	carnitine metabolic process (GO:0009437)	0.0002415	0.006832	-3.03	25.27
6	ketone body biosynthetic process (GO:0046951)	0.0001322	0.005819	-2.74	24.49
7	sarcomere organization (GO:0045214)	0.00003270	0.002158	-2.30	23.73
8	negative regulation of potassium ion transmembrane transport (GO:1901380)	0.0001651	0.006538	-2.49	21.70
9	positive regulation of potassium ion transmembrane transporter activity (GO:1901018)	0.0002851	0.007056	-2.60	21.26
10	negative regulation of potassium ion transmembrane transporter activity (GO:1901017)	0.0001322	0.005819	-2.28	20.39

Cellular components

Index	Name	P-value	Adjusted p-value	Z-score	Combined score
1	mitochondrion (GO:0005739)	3.668e-9	1.575e-7	-1.85	36.00
2	mitochondrial matrix (GO:0005759)	6.300e-9	1.575e-7	-1.19	22.49
3	sarcoplasmic reticulum (GO:0016529)	0.00002684	0.0004087	-2.10	22.11
4	sarcoplasm (GO:0016528)	0.00003270	0.0004087	-2.09	21.57
5	contractile fiber (GO:0043292)	0.001261	0.007882	-2.12	14.13
6	striated muscle thin filament (GO:0005865)	0.0006885	0.005628	-1.93	14.02
7	microbody (GO:0042579)	0.0007879	0.005628	-1.87	13.37
8	peroxisome (GO:0005777)	0.0007879	0.005628	-1.76	12.56
9	junctional sarcoplasmic reticulum membrane (GO:0014701)	0.01933	0.07550	-3.11	12.29
10	pseudopodium (GO:0031143)	0.01933	0.07550	-2.88	11.35

Fig. S1A

Consensus gene set name: HumanSkelMusc_1DayOrLessAfterExercise_v_Before

Number of gene sets drawn from: 18 (9 up, 9 down)

EnrichR test carried out on consensus proportion of: 30% Same: 141 genes (13.08%)

Biological processes

Index	Name	P-value	Adjusted p-value	Z-score	Combined score
1	positive regulation of fatty acid oxidation (GO:0046321)	0.00007203	0.01056	-3.14	29.93
2	regulation of epithelial cell proliferation (GO:0050678)	0.00001274	0.003364	-2.23	25.14
3	regulation of fatty acid oxidation (GO:0046320)	0.000002243	0.001480	-1.78	23.12
4	positive regulation of cell migration by vascular endothelial growth factor signaling pathway (GO:0038089)	0.001344	0.04327	-3.48	22.98
5	positive regulation of transcription from RNA polymerase II promoter (GO:0045944)	0.000008325	0.002747	-1.89	22.15
6	cellular response to cold (GO:0070417)	0.001013	0.03931	-3.15	21.75
7	photoperiodism (GO:0009648)	0.0004788	0.02633	-2.75	20.99
8	cell migration involved in sprouting angiogenesis (GO:0002042)	0.0001467	0.01383	-2.33	20.57
9	negative regulation of glucocorticoid receptor signaling pathway (GO:2000323)	0.001013	0.03931	-2.84	19.58
10	negative regulation of transcription, DNA-templated (GO:0045892)	0.00001725	0.003794	-1.76	19.28

Cellular components

Cell components: none significant

Fig. S1B

Consensus gene set name: MurineMyotube_9hOrLessDiff_v_Undiff
Number of gene sets drawn from: 18 (9 up, 9 down)
EnrichR test carried out on consensus proportion of: 30% Same: 68 genes (4.73%)

Biological processes

Index	Name	P-value	Adjusted p-value	Z-score	Combined score
1	negative regulation of neuron differentiation (GO:0045665)	0.000002759	0.002475	-2.14	27.35
2	positive regulation of extrinsic apoptotic signaling pathway via death domain receptors (GO:1902043)	0.0002366	0.01307	-3.24	27.01
3	regulation of wound healing (GO:0061041)	0.000005910	0.002651	-1.87	22.49
4	negative regulation of cell adhesion mediated by integrin (GO:0033629)	0.0003148	0.01506	-2.70	21.73
5	regulation of blood coagulation (GO:0030193)	0.0001578	0.01011	-2.43	21.25
6	positive regulation of transcription from RNA polymerase II promoter (GO:0045944)	0.00002579	0.004489	-1.89	19.99
7	positive regulation of hemostasis (GO:1900048)	0.0005036	0.01506	-2.60	19.73
8	blood vessel endothelial cell migration (GO:0043534)	0.00003503	0.004489	-1.88	19.28
9	negative regulation of fibrinolysis (GO:0051918)	0.0005036	0.01506	-2.51	19.06
10	positive regulation of wound healing (GO:0090303)	0.0001037	0.007752	-2.07	19.03

Cellular components

Index	Name	P-value	Adjusted p-value	Z-score	Combined score
1	nuclear chromatin (GO:0000790)	0.0002241	0.006050	-2.01	16.89
2	chromatin (GO:0000785)	0.00006629	0.003580	-1.70	16.34
3	euchromatin (GO:0000791)	0.002518	0.02720	-2.59	15.50
4	platelet alpha granule lumen (GO:0031093)	0.001609	0.02720	-1.97	12.67
5	platelet alpha granule (GO:0031091)	0.003689	0.03320	-1.59	8.91
6	npBAF complex (GO:0071564)	0.04006	0.2262	-2.59	8.34
7	nuclear chromosome part (GO:0044454)	0.002174	0.02720	-1.22	7.49
8	platelet dense granule lumen (GO:0031089)	0.04982	0.2262	-2.38	7.14
9	nuclear euchromatin (GO:0005719)	0.06269	0.2262	-2.44	6.75
10	SWI/SNF complex (GO:0016514)	0.05305	0.2262	-2.25	6.60

Fig. S1C

Consensus gene set name: MurineMyotube_12to24hDiff_v_Undiff
Number of gene sets drawn from: 14 (7 up, 7 down)
EnrichR test carried out on consensus proportion of: 50% Same: 101 genes (7.57%)

Biological processes

Index	Name	P-value	Adjusted p-value	Z-score	Combined score
1	regulation of transcription involved in G1/S transition of mitotic cell cycle (GO:0000083)	3.877e-9	8.149e-7	-1.91	37.02
2	DNA metabolic process (GO:0006259)	4.692e-12	2.466e-9	-1.36	35.55
3	G1/S transition of mitotic cell cycle (GO:0000082)	2.089e-13	2.196e-10	-1.19	34.88
4	positive regulation of chromosome segregation (GO:0051984)	0.00002661	0.001997	-3.06	32.28
5	cell cycle G1/S phase transition (GO:0044843)	3.907e-11	1.369e-8	-1.20	28.86
6	DNA replication (GO:0006260)	1.796e-7	0.00002360	-1.55	24.07
7	mitotic cell cycle phase transition (GO:0044772)	1.299e-9	3.414e-7	-1.17	23.91
8	endoplasmic reticulum calcium ion homeostasis (GO:0032469)	0.0001142	0.006669	-2.50	22.73
9	mitotic spindle organization (GO:0007052)	1.017e-7	0.00001527	-1.39	22.33
10	microtubule cytoskeleton organization involved in mitosis (GO:1902850)	0.000003094	0.0003613	-1.64	20.84

Cellular components

Index	Name	P-value	Adjusted p-value	Z-score	Combined score
1	alpha DNA polymerase:primase complex (GO:0005658)	0.000004311	0.0001089	-3.69	45.63
2	nuclear chromosome part (GO:0044454)	1.234e-9	1.246e-7	-1.25	25.64
3	sarcoplasmic reticulum (GO:0016529)	0.00001518	0.0003034	-2.08	23.09
4	nuclear replisome (GO:0043601)	0.00008074	0.001165	-2.27	21.36
5	nuclear chromosome (GO:0000228)	0.0001484	0.001665	-2.11	18.60
6	spindle (GO:0005819)	4.488e-7	0.00002267	-1.18	17.20
7	calcium channel complex (GO:0034704)	0.0002652	0.002678	-1.96	16.12
8	replication fork (GO:0005657)	0.0003348	0.003074	-1.95	15.60
9	chromosome, telomeric region (GO:0000781)	0.000003326	0.0001089	-1.23	15.53
10	sarcoplasm (GO:0016528)	0.0005574	0.004692	-2.01	15.09

Fig. S1D

Consensus gene set name: MurineMyotube_2orMoreDaysDiff_v_Undiff
Number of gene sets drawn from: 38 (19 up, 19 down)
EnrichR test carried out on consensus proportion of: 30% Same: 188 genes (5.61%)

Biological processes

Index	Name	P-value	Adjusted p-value	Z-score	Combined score
1	muscle contraction (GO:0006936)	5.931e-44	7.787e-41	-1.18	117.05
2	muscle filament sliding (GO:0030049)	7.512e-27	3.288e-24	-1.80	108.01
3	actin-myosin filament sliding (GO:0033275)	7.512e-27	3.288e-24	-1.66	100.05
4	skeletal muscle contraction (GO:0003009)	2.668e-18	5.005e-16	-2.15	86.86
5	myofibril assembly (GO:0030239)	3.369e-21	8.848e-19	-1.82	85.67
6	sarcomere organization (GO:0045214)	6.399e-17	1.050e-14	-2.29	85.55
7	striated muscle contraction (GO:0006941)	3.676e-24	1.207e-21	-1.23	66.24
8	regulation of calcium ion transmembrane transport (GO:1903169)	9.622e-12	9.024e-10	-2.25	57.10
9	heart contraction (GO:0060047)	6.048e-20	1.324e-17	-1.28	56.58
10	cardiac muscle contraction (GO:0060048)	5.040e-16	7.353e-14	-1.42	49.91

Cellular components

Index	Name	P-value	Adjusted p-value	Z-score	Combined score
1	alpha DNA polymerase:primase complex (GO:0005658)	0.000004311	0.0001089	-3.69	45.63
2	nuclear chromosome part (GO:0044454)	1.234e-9	1.246e-7	-1.25	25.64
3	sarcoplasmic reticulum (GO:0016529)	0.00001518	0.0003034	-2.08	23.09
4	nuclear replisome (GO:0043601)	0.00008074	0.001165	-2.27	21.36
5	nuclear chromosome (GO:0000228)	0.0001484	0.001665	-2.11	18.60
6	spindle (GO:0005819)	4.488e-7	0.00002267	-1.18	17.20
7	calcium channel complex (GO:0034704)	0.0002652	0.002678	-1.96	16.12
8	replication fork (GO:0005657)	0.0003348	0.003074	-1.95	15.60
9	chromosome, telomeric region (GO:0000781)	0.000003326	0.0001089	-1.23	15.53
10	sarcoplasm (GO:0016528)	0.0005574	0.004692	-2.01	15.09

Fig. S1E

Consensus gene set name: HumanSkelMusc_DMD_v_Healthy
Number of gene sets drawn from: 8 (4 up, 4 down)
EnrichR test carried out on consensus proportion of: 50% Upregulated: 84 genes (15.47%)

Biological processes

Index	Name	P-value	Adjusted p-value	Z-score	Combined score
1	extracellular matrix organization (GO:0030198)	7.410e-13	6.321e-10	-1.65	45.99
2	collagen fibril organization (GO:0030199)	1.520e-7	0.00006483	-1.58	24.83
3	positive regulation of smooth muscle cell migration (GO:0014911)	0.0009354	0.04602	-3.49	24.31
4	glycosaminoglycan catabolic process (GO:0006027)	0.000004086	0.0005810	-1.94	24.09
5	skeletal muscle contraction (GO:0003009)	0.00006597	0.008039	-2.15	20.66
6	regulation of sterol transport (GO:0032371)	0.0004801	0.03150	-2.68	20.44
7	protein complex subunit organization (GO:0071822)	0.000001387	0.0003944	-1.45	19.51
8	skeletal system development (GO:0001501)	0.000002834	0.0005038	-1.47	18.80
9	skin development (GO:0043588)	0.000002953	0.0005038	-1.35	17.22
10	regulation of T cell proliferation (GO:0042129)	0.0001428	0.01218	-1.85	16.41

Cellular components

Index	Name	P-value	Adjusted p-value	Z-score	Combined score
1	vacuolar lumen (GO:0005775)	2.630e-8	6.663e-7	-2.22	38.74
2	lysosomal lumen (GO:0043202)	3.075e-9	2.337e-7	-1.73	33.86
3	endoplasmic reticulum lumen (GO:0005788)	1.788e-8	6.663e-7	-1.54	27.41
4	actin-based cell projection (GO:0098858)	0.00001214	0.0001571	-1.92	21.76
5	lytic vacuole (GO:0000323)	0.00001241	0.0001571	-1.72	19.39
6	lysosome (GO:0005764)	0.000001545	0.00002936	-1.15	15.36
7	endolysosome (GO:0036019)	0.003475	0.02641	-2.32	13.13
8	extrinsic component of external side of plasma membrane (GO:0031232)	0.03312	0.09706	-3.47	11.84
9	filopodium (GO:0030175)	0.002158	0.01822	-1.91	11.75
10	microvillus (GO:0005902)	0.0007813	0.008483	-1.59	11.38

Fig. S1F

Consensus gene set name: HumanSkelMusc_DMD_v_Healthy
Number of gene sets drawn from: 8 (4 up, 4 down)
EnrichR test carried out on consensus proportion of: 50% Downregulated: 21 genes (7.53%)

Biological processes

Index	Name	P-value	Adjusted p-value	Z-score	Combined score
1	regulation of sodium ion transmembrane transport (GO:1902305)	0.000004400	0.001067	-1.97	24.27
2	regulation of ventricular cardiac muscle cell membrane repolarization (GO:0060307)	0.0001094	0.007381	-2.40	21.87
3	regulation of sodium ion transmembrane transporter activity (GO:2000649)	0.000008220	0.001067	-1.75	20.50
4	regulation of cardiac muscle cell membrane repolarization (GO:0099623)	0.0001776	0.007994	-2.23	19.30
5	dopamine catabolic process (GO:0042420)	0.007328	0.07261	-3.62	17.82
6	cardiac muscle cell action potential (GO:0086001)	0.0004487	0.01346	-2.14	16.51
7	regulation of membrane depolarization (GO:0003254)	0.0001415	0.007639	-1.79	15.91
8	mannose metabolic process (GO:0006013)	0.008371	0.07261	-3.31	15.81
9	regulation of skeletal muscle contraction (GO:0014819)	0.007328	0.07261	-3.12	15.36
10	catecholamine catabolic process (GO:0042424)	0.009412	0.07261	-3.08	14.37

Cellular components

Index	Name	P-value	Adjusted p-value	Z-score	Combined score
1	dystrophin-associated glycoprotein complex (GO:0016010)	0.0001094	0.004265	-2.66	24.27
2	filopodium membrane (GO:0031527)	0.01357	0.1159	-2.59	11.14
3	node of Ranvier (GO:0033268)	0.01357	0.1159	-2.57	11.04
4	DNA-directed RNA polymerase complex (GO:0000428)	0.01771	0.1159	-2.20	8.87
5	DNA-directed RNA polymerase II, core complex (GO:0005665)	0.01874	0.1159	-2.14	8.50
6	contractile fiber (GO:0043292)	0.02798	0.1245	-2.12	7.57
7	striated muscle thin filament (GO:0005865)	0.02080	0.1159	-1.90	7.37
8	myofibril (GO:0030016)	0.02901	0.1245	-2.00	7.09
9	mitochondrial matrix (GO:0005759)	0.003952	0.07706	-1.19	6.59
10	intercalated disc (GO:0014704)	0.03308	0.1245	-1.65	5.61

Fig. S1G

Consensus gene set name: MurineSkelMusc6WkOrOlder_Mdx_v_Healthy
Number of gene sets drawn from: 36 (18 up, 18 down)
EnrichR test carried out on consensus proportion of: 30% Up: 73 genes (5.07%)

Biological processes

Index	Name	P-value	Adjusted p-value	Z-score	Combined score
1	extracellular matrix organization (GO:0030198)	9.651e-14	7.055e-11	-1.65	49.34
2	sarcomere organization (GO:0045214)	0.000005436	0.0005676	-2.30	27.83
3	myofibril assembly (GO:0030239)	8.542e-7	0.0003122	-1.82	25.45
4	negative regulation of type 2 immune response (GO:0002829)	0.0003627	0.01061	-3.14	24.88
5	skeletal muscle contraction (GO:0003009)	0.00004335	0.002437	-2.14	21.53
6	positive regulation of calcium ion import (GO:0090280)	0.0007075	0.01398	-2.80	20.31
7	positive regulation of myoblast fusion (GO:1901741)	0.0008470	0.01510	-2.86	20.25
8	muscle filament sliding (GO:0030049)	0.00001220	0.0008104	-1.79	20.25
9	regulation of immune effector process (GO:0002697)	0.0008318	0.01510	-2.82	19.98
10	positive regulation of interleukin-2 production (GO:0032743)	0.00005919	0.002810	-2.05	19.92

Cellular components

Index	Name	P-value	Adjusted p-value	Z-score	Combined score
1	vacuolar lumen (GO:0005775)	0.0003158	0.008210	-2.22	17.89
2	endoplasmic reticulum lumen (GO:0005788)	0.00005897	0.004600	-1.55	15.09
3	lysosomal lumen (GO:0043202)	0.0002892	0.008210	-1.72	14.00
4	endolysosome (GO:0036019)	0.002638	0.01871	-2.31	13.73
5	filopodium (GO:0030175)	0.001442	0.01607	-1.95	12.74
6	extrinsic component of external side of plasma membrane (GO:0031232)	0.02883	0.08659	-3.47	12.32
7	platelet alpha granule lumen (GO:0031093)	0.001972	0.01866	-1.91	11.88
8	endosome lumen (GO:0031904)	0.003734	0.02240	-2.06	11.49
9	actin-based cell projection (GO:0098858)	0.002232	0.01866	-1.86	11.35
10	striated muscle thin filament (GO:0005865)	0.002393	0.01866	-1.87	11.29

Fig. S1H

Consensus gene set name: HumanOrMurineSkelMusc_Dysferlinopathy_v_Control
Number of gene sets drawn from: 12 (6 up, 6 down)
EnrichR test carried out on consensus proportion of: 30% Same: 188 genes (26.97%)

Biological processes

Index	Name	P-value	Adjusted p-value	Z-score	Combined score
1	neutrophil mediated immunity (GO:0002446)	5.764e-12	1.025e-8	-1.95	50.38
2	neutrophil degranulation (GO:0043312)	2.624e-11	1.848e-8	-2.02	49.12
3	regulation of protein modification by small protein conjugation or removal (GO:1903320)	0.000009519	0.002419	-2.96	34.19
4	regulation of chemokine biosynthetic process (GO:0045073)	0.00004421	0.004916	-3.30	33.08
5	neutrophil activation involved in immune response (GO:0002283)	3.117e-11	1.848e-8	-1.25	30.23
6	cellular response to oxidative stress (GO:0034599)	0.00001404	0.002906	-2.03	22.64
7	miRNA mediated inhibition of translation (GO:0035278)	0.0001689	0.01128	-2.43	21.08
8	negative regulation of complement activation (GO:0045916)	0.001789	0.05118	-3.30	20.90
9	regulation of epidermal cell differentiation (GO:0045604)	0.00003993	0.004736	-2.05	20.81
10	positive regulation of tumor necrosis factor-mediated signaling pathway (GO:1903265)	0.002371	0.05144	-3.34	20.16

Cellular components

Index	Name	P-value	Adjusted p-value	Z-score	Combined score
1	vacuolar lumen (GO:0005775)	4.512e-9	6.949e-7	-2.26	43.34
2	lysosomal lumen (GO:0043202)	9.059e-9	6.976e-7	-1.72	31.81
3	endosome lumen (GO:0031904)	0.00008196	0.003155	-2.12	19.96
4	ficolin-1-rich granule lumen (GO:1904813)	0.0001730	0.004838	-2.21	19.14
5	endolysosome (GO:0036019)	0.0009596	0.01642	-2.33	16.17
6	ficolin-1-rich granule (GO:0101002)	0.00001034	0.0005310	-1.27	14.57
7	specific granule membrane (GO:0035579)	0.001768	0.02325	-1.75	11.11
8	secondary lysosome (GO:0005767)	0.007425	0.07623	-2.19	10.75
9	pericentriolar material (GO:0000242)	0.01089	0.09869	-2.36	10.68
10	secretory granule lumen (GO:0034774)	0.0002199	0.004838	-1.26	10.61

Fig. S1I

Consensus gene set name: HumanSkelMusc_Aging_v_Young
Number of gene sets drawn from: 16 (8 up, 8 down)
EnrichR test carried out on consensus proportion of: 30% Same: 34 genes (1.82%)

Biological processes

Index	Name	P-value	Adjusted p-value	Z-score	Combined score
1	positive regulation of neuron apoptotic process (GO:0043525)	0.0005228	0.08164	-2.36	17.83
2	mitochondrial ATP synthesis coupled proton transport (GO:0042776)	0.03676	0.1107	-4.90	16.18
3	positive regulation of transcription from RNA polymerase II promoter (GO:0045944)	0.0004773	0.08164	-1.90	14.52
4	negative regulation of T cell proliferation (GO:0042130)	0.0008212	0.08164	-2.04	14.50
5	regulation of dendritic spine maintenance (GO:1902950)	0.01352	0.1061	-3.26	14.02
6	negative regulation of oligodendrocyte differentiation (GO:0048715)	0.01184	0.1061	-3.16	14.01
7	gluconeogenesis (GO:0006094)	0.002314	0.08164	-2.12	12.90
8	negative regulation of glial cell differentiation (GO:0045686)	0.01520	0.1061	-3.07	12.86
9	positive regulation of neuron death (GO:1901216)	0.001998	0.08164	-2.04	12.70
10	neuron projection maintenance (GO:1990535)	0.01184	0.1061	-2.86	12.67

Cellular components

Cell components: none significant

Fig. S1J

Consensus gene set name: HumanSkelMusc_8wkOrMoreAfterResistanceTraining_v_Before
Number of gene sets drawn from: 12 (6 up, 6 down)
EnrichR test carried out on consensus proportion of: 30% Upregulated: 24 genes (11.54%)

Biological processes

Index	Name	P-value	Adjusted p-value	Z-score	Combined score
1	extracellular matrix organization (GO:0030198)	5.665e-14	1.314e-11	-1.65	50.22
2	basement membrane organization (GO:0071711)	0.00004943	0.002556	-2.84	28.18
3	collagen fibril organization (GO:0030199)	4.279e-8	0.000004964	-1.58	26.84
4	protein complex subunit organization (GO:0071822)	2.516e-7	0.00001946	-1.45	21.98
5	regulation of angiogenesis (GO:0045765)	0.00005608	0.002556	-1.77	17.36
6	spinal cord motor neuron differentiation (GO:0021522)	0.008371	0.06509	-3.19	15.24
7	collagen biosynthetic process (GO:0032964)	0.008371	0.06509	-3.07	14.70
8	positive regulation of epithelial to mesenchymal transition (GO:0010718)	0.001100	0.02320	-2.12	14.48
9	coronary vasculature development (GO:0060976)	0.008371	0.06509	-2.92	13.97
10	negative regulation of cAMP-mediated signaling (GO:0043951)	0.008371	0.06509	-2.90	13.86

Cellular components

Index	Name	P-value	Adjusted p-value	Z-score	Combined score
1	endoplasmic reticulum lumen (GO:0005788)	6.421e-7	0.00001413	-1.55	22.10
2	platelet alpha granule membrane (GO:0031092)	0.0002087	0.002296	-2.15	18.20
3	endocytic vesicle lumen (GO:0071682)	0.02257	0.1241	-2.58	9.77
4	platelet alpha granule (GO:0031091)	0.005295	0.03883	-1.62	8.49
5	sarcoplasmic reticulum (GO:0016529)	0.03541	0.1383	-2.08	6.95
6	sarcoplasm (GO:0016528)	0.03772	0.1383	-2.07	6.79
7	nuclear matrix (GO:0016363)	0.06961	0.1813	-2.13	5.68
8	platelet alpha granule lumen (GO:0031093)	0.07853	0.1813	-1.90	4.82
9	specific granule membrane (GO:0035579)	0.1048	0.1922	-1.74	3.93
10	ficolin-1-rich granule membrane (GO:0101003)	0.07185	0.1813	-1.31	3.46

Fig. S1K

Consensus gene set name: MurineSkelMusc_12orMoreMonths_v_1to5Months
Number of gene sets drawn from: 8 (4 up, 4 down)
EnrichR test carried out on consensus proportion of: 30% Same: 24 genes (3.76%)

Biological processes

Index	Name	P-value	Adjusted p-value	Z-score	Combined score
1	collagen fibril organization (GO:0030199)	4.279e-8	0.00001018	-1.58	26.85
2	protein complex subunit organization (GO:0071822)	2.516e-7	0.00002994	-1.45	21.99
3	extracellular matrix organization (GO:0030198)	0.000006848	0.0005433	-1.64	19.55
4	intermediate filament bundle assembly (GO:0045110)	0.008371	0.07310	-3.51	16.77
5	regulation of isotype switching (GO:0045191)	0.009561	0.07310	-3.55	16.50
6	bundle of His cell to Purkinje myocyte communication (GO:0086069)	0.01075	0.07310	-3.61	16.35
7	cardiac muscle cell-cardiac muscle cell adhesion (GO:0086042)	0.009561	0.07310	-3.30	15.37
8	bundle of His cell-Purkinje myocyte adhesion involved in cell communication (GO:0086073)	0.008371	0.07310	-3.19	15.24
9	iron ion import (GO:0097286)	0.008371	0.07310	-3.12	14.93
10	insulin secretion involved in cellular response to glucose stimulus (GO:0035773)	0.008371	0.07310	-3.08	14.74

Cellular components

Index	Name	P-value	Adjusted p-value	Z-score	Combined score
1	endoplasmic reticulum lumen (GO:0005788)	0.00001515	0.0005606	-1.55	17.20
2	recycling endosome (GO:0055037)	0.0003889	0.007194	-1.27	10.00
3	HFE-transferrin receptor complex (GO:1990712)	0.01075	0.1326	-3.96	17.96
4	microtubule organizing center (GO:0005815)	0.02219	0.1886	-2.07	7.90
5	pre-autophagosomal structure (GO:0000407)	0.02842	0.1886	-1.82	6.49
6	Cajal body (GO:0015030)	0.04119	0.1886	-1.85	5.91
7	intercalated disc (GO:0014704)	0.03772	0.1886	-1.66	5.45
8	intermediate filament (GO:0005882)	0.05493	0.1886	-1.50	4.36
9	lytic vacuole membrane (GO:0098852)	0.03177	0.1886	-1.19	4.11
10	recycling endosome membrane (GO:0055038)	0.05607	0.1886	-1.40	4.03

Fig. S1L

Consensus gene set name: HumanSkelMusc_Type2_Diabetes_v_Healthy
Number of gene sets drawn from: 10 (5 up, 5 down)
EnrichR test carried out on consensus proportion of:

No overlapping genes found

Fig. S1M

Consensus gene set name: MurineSkelMusc_Calpainopathy_v_Healthy
Number of gene sets drawn from: 14 (7 up, 7 down)
EnrichR test carried out on consensus proportion of: 30% Same: 12 genes (1.22%)

Biological processes **Cellular components**

Nothing significant

Fig. S1N

Consensus gene set name: AnySpeciesSkelMusc_AtrophyDisuseOrInactivated_v_Control

Number of gene sets drawn from: 28 (14 up, 14 down)

Only 4 overlapping genes found (down in 30%; 0.29% of total)

Fig. S1O
